# Supplementary material for: Identification of photoperiod-induced specific miRNAs in the adrenal glands of Sunite sheep (Ovis aries)
Source: Front Vet Sci. 2022 Jul 22;9:888207. doi: 10.3389/fvets.2022.888207 (PMC9354845; doi:10.3389/fvets.2022.888207)
Supplement: Supplementary file 1 [file Data_Sheet_1.ZIP › Supplementary file/miRDeep2 file.zip!/miRDeep2 file/Know_Structure/LP42A3/oar-mir-10a.pdf]

miRBase precursor : oar-mir-10a
Total read count : 119105
oar-miR-10a read count : 118392
remaining reads : 713

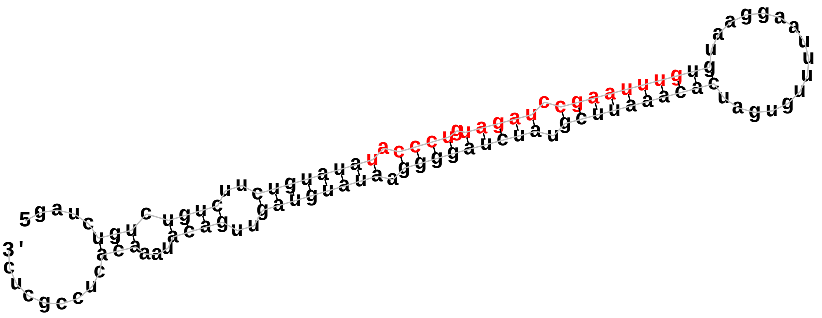

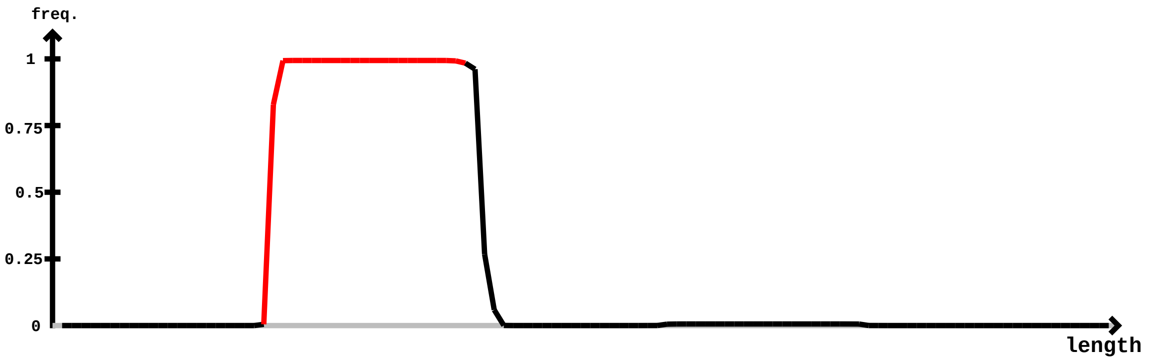


```
                        oar-miR-10a
5'- gaucugucugucuucuguauauacccuguagauccgaauuuguguaaggaauuuugugaucacaaauucguaucuaggggaauauguaguugacauaaacacuccgcuc -3'   exp
    ....(((.((((..((((((((.((((.(((((.((((((((((...............)))))))))).)))))))))).))))))))..))))...)))....... reads  mm  sample
    ..ucugucugucuucuguaua........................................................................................ 1      0   seq
    ...................auacccuguagauccgaauu...................................................................... 4      0   seq
    ...................auacccuguagauccgaauuu..................................................................... 34     0   seq
    ...................auacccuguagauccgaauuug.................................................................... 65     0   seq
    ...................auacccuguagauccgaauuugu................................................................... 421    0   seq
    ...................auacccuguagauccgaauuugug.................................................................. 36     0   seq
    ...................auacccuguagauccgaauuugugu................................................................. 3      0   seq
    ....................uacccuguagauccgaa........................................................................ 3      0   seq
    ....................uacccuguagauccgaau....................................................................... 13     0   seq
    ....................uacccuguagauccgaauu...................................................................... 147    0   seq
    ....................uacccuguagauccgaauuu..................................................................... 838    0   seq
    ....................uacccuguagauccgaauuug.................................................................... 2325   0   seq
    ....................uacccuguagauccgaauuugu................................................................... 73296  0   seq
    ....................uacccuguagauccgaauuugug.................................................................. 17454  0   seq
    ....................uacccuguagauccgaauuugugu................................................................. 4022   0   seq
    ....................uacccuguagauccgaauuugugua................................................................ 21     0   seq
    .....................acccuguagauccgaau....................................................................... 1      0   seq
    .....................acccuguagauccgaauu...................................................................... 20     0   seq
    .....................acccuguagauccgaauuu..................................................................... 135    0   seq
    .....................acccuguagauccgaauuug.................................................................... 305    0   seq
    .....................acccuguagauccgaauuugu................................................................... 8888   0   seq
    .....................acccuguagauccgaauuugug.................................................................. 7351   0   seq
    .....................acccuguagauccgaauuugugu................................................................. 2923   0   seq
    .....................acccuguagauccgaauuugugua................................................................ 18     0   seq
    .....................acccuguagauccgaauuuguguaa............................................................... 1      0   seq
    ......................cccuguagauccgaauuu..................................................................... 1      0   seq
    ......................cccuguagauccgaauuug.................................................................... 1      0   seq
    ......................cccuguagauccgaauuugu................................................................... 15     0   seq
    ......................cccuguagauccgaauuugug.................................................................. 26     0   seq
    ......................cccuguagauccgaauuugugu................................................................. 19     0   seq
    ......................cccuguagauccgaauuugugua................................................................ 1      0   seq
    .......................ccuguagauccgaauuugu................................................................... 1      0   seq
    .......................ccuguagauccgaauuugug.................................................................. 2      0   seq
    .......................ccuguagauccgaauuugugu................................................................. 1      0   seq
    ........................cuguagauccgaauuugu................................................................... 1      0   seq
```

oar-miR-10a

gaucugucugucuucuguauauacccuguagauccgaauuuguguaaggaauuuugugaucacaaauucguaucuaggggaauauguaguugacauaaacacuccgcuc			
....uaaggaauuuugugauca...	1	0	seq
....ucacaaauucguaucuagggga............................	1	0	seq
....cacaaauucguaucuagggga............................	1	0	seq
....cacaaauucguaucuaggggaa...........................	4	0	seq
....cacaaauucguaucuaggggaau..........................	1	0	seq
....acaaauucguaucuagggga............................	2	0	seq
....acaaauucguaucuaggggaa...........................	1	0	seq
....acaaauucguaucuaggggaau..........................	16	0	seq
....caaauucguaucuagggg.............................	1	0	seq
....caaauucguaucuagggga............................	7	0	seq
....caaauucguaucuaggggaa...........................	9	0	seq
....caaauucguaucuaggggaau..........................	576	0	seq
....caaauucguaucuaggggaaua.........................	8	0	seq
....caaauucguaucuaggggaauau........................	1	0	seq
....aaauucguaucuaggggaa...........................	2	0	seq
....aaauucguaucuaggggaau..........................	69	0	seq
....aaauucguaucuaggggaaua.........................	3	0	seq
....aaauucguaucuaggggaauau........................	3	0	seq
....aauucguaucuaggggaau..........................	2	0	seq
....aauucguaucuaggggaauau........................	3	0	seq
....uguaguugacauaaacacucc....	1	0	seq
